# Understanding urban concentration of complex manufacturing activities in China

**DOI:** 10.1371/journal.pone.0278469

**Published:** 2023-03-16

**Authors:** Linzhuo Li, Nannan Zhao

**Affiliations:** 1 Department of Sociology, Zhejiang University, Hangzhou, China; 2 Culture and Knowledge Lab, Zhejiang University, Hangzhou, China; University of Sargodha, PAKISTAN

## Abstract

The increasing prominence of urban scaling laws highlights the importance of a systematic understanding of the variational scaling rates for different economic activities. In this article, we utilize several datasets to provide the first systematic investigation of the urban scaling of manufacturing industries in China. Most existing literature assumes that the divergence in urban scaling can be explained by returns to agglomeration, with a few exceptions instead highlighting the role of knowledge complexity or a mixture of both. Our main purpose in this paper is to explain the inter-sector variation of urban scaling rates. In doing this, we provide a clearer approach to demonstrating the relations between urban scaling, returns to agglomeration, and knowledge complexity. Our findings are twofold. First, after uncovering the scaling rates (denoted as *urban concentration*) and returns to agglomeration (denoted as *urban productivity*) for each sub-manufacturing sector, we prove that, rather than being a positive predictor, returns to agglomeration is slightly negatively associated with urban scaling rates. This finding reveals that urban concentration of manufacturing may not simply be a natural consequence driven by the maximization of performance. We also show that this result of the manufacturing system contrasts with what would be found in other pure knowledge systems such as patents. Secondly, we measure the complexity for each sector and demonstrate that the variation of urban concentration can be largely explained by their complexity, consistent with the knowledge complexity perspective. Specifically, complex manufacturing sectors are found to concentrate more in large cities than less complex sectors in China. This result provides support for the view that the growth of complex activities hinges more on diversity than on efficiency. The findings above can greatly reduce the current level of ambiguity associated with urban scaling, returns to agglomeration and complexity, and have important policy implications for urban planners, highlighting the significance of a more balanced and diversified configuration of urban productive activities for the growth of innovation economy.

## Introduction

The concentration of economic activities is central to the growth of cities. Despite being a traditional topic [1–5], the emergence of a prominent “new science of cities” have highlighted urban scaling laws as simple yet powerful ways for understanding the spatial patterns of a wide variety of urban activities [6–8]. Urban scaling laws suggest that most social-economic and infrastructural variables display scaling behaviors with the growth of city population, just like living organisms in biology [9, 10]. Cumulated research in this area has provided enormous wisdom for urban theories and urban planning [11–15]. In this paper, we enrich the scaling research by providing the first systematic analysis of urban scaling of find-grained manufacturing activities in China—currently the world’s biggest manufacturer. In particular, we seek to further our understanding of urban scaling by examining and distinguishing its relations with two important notions—returns to agglomeration and knowledge complexity.

As is widely known, one of the major characteristics of China’s fast urbanization over the last few decades is the associated urban agglomeration of manufacturing industries. Just like elsewhere in the world, and especially in the past few decades of fast urbanization, the agglomeration of manufacturing industries increases steadily [16] and has become an important condition for the country’s participation in the global value chain and its surprising growth into the world’s largest manufacturer [17–19]. Understanding the spatial concentration pattern of manufacturing activities in China has therefore long been an important question.

A rich body of literature on the degree and difference of manufacturing agglomeration in China has been developed. Among them, there are two major directions. The first direction mainly concerns spatial dependence or relatedness. The key point of this strand is that industrial activities are spatially correlated and therefore supporting industries tend to facilitate the growth of each other into agglomerations of related industries. For instance, Li and colleagues [20] use location quotient and spatial autocorrelation methods to examine the industrial agglomeration patterns of Hebei province in China and find evidence of uneven agglomeration for many manufacturing sectors. Ye and collaborators [19] find evidence of a regional collaborative “division of labor” between highly-developed cities and lowly-economic-developed cities, which affects industrial agglomeration. The second strand concerns more about social-political factors such as ownership, regional planning, and globalization. For example, using census data, Lu and colleagues [21] showed that China’s non-public-owned industrial establishments are more spatially concentrated than public-own ones. Others [22] found that industries driven by global forces are more concentrated in China. Lu and Tao [16] show that local protectionism obstructs while Marshallian externalities facilitate the spatial concentration of manufacturing industries.

Despite their richness in the details of relational features or locally specific factors of manufacturing agglomeration, providing a concise yet general explanation of the variation in concentration remains a challenge. Few of these studies choose to examine the agglomeration of various manufacturing activities from the more universal and comparative perspective—urban scaling. We argue that such a gap should be filled, as urban scaling is increasingly recognized as a major driving force for the spatial cumulation of activities in a wide variety of economies including China [23–27]. Uncovering the detailed scaling structure of China’s manufacturing industries, therefore, is of both theoretical importance and empirical relevance.

Our analysis is threefold. First, we investigate the urban scaling structure (denoted as *urban concentration*) for all sub-manufacturing industries in China. This investigation remains important because most existing analyses of urban scaling tend to treat manufacturing activities as a whole without looking at the more fine-grained subsector levels. Therefore, despite the general wisdom that manufacturing activities scale super-linearly, it remains unknown whether urban scaling still holds for sub-sectors and if so, what the scaling structure of these sub-sectors would look like. In the paper, we find a huge variation in scaling behaviors for different sub-sectors. It is important to explain these differences.

Secondly, we measure each sector’s returns to agglomeration (denoted as *urban productivity*). The measure is similar to scale elasticity in industrial economics but applied to the urban context. This inquiry allows us to re-examine the performance assumption of urban scaling. After linking it with urban concentration, we found that, interestingly, those industries with greater urban concentration are not those with higher returns to agglomeration. Rather, the relationship is slightly negative. Therefore, it challenges the view that urban scaling rates can be explained mainly by performance.

Thirdly, we ask, if returns to agglomeration fail to explain urban scaling, then what might be the major factor? We follow recent advances in economic complexity to test the effect of complexity on the scaling of industrial employment and identify a strong positive effect in our data. This result provides supportive evidence of the linkage between urban diversity rather than urban productivity for super-linear scaling. It is important because even studies that previously adopted the view of complexity often assume contradicting logic and mix up mechanisms of returns to agglomeration with that of knowledge complexity.

Essentially, our approach distinguishes between urban input scaling and output scaling. The overall findings complicate performance-based interpretations, especially about the urban scaling of innovative economy dominant in the existing literature. We conclude by discussing the policy implications of our findings for urban planning and the innovation economy.

## Literature review

Why do some Chinese manufacturing industries concentrate more than others in large cities? Explaining such diverging scaling behavior of various social-economic units has long been an important yet challenging task. Most recent studies often hint on the linkage between the property of scaling rate with (increasing) benefits or returns, usually knowns as returns to scale or agglomeration [23, 28–30]. The logic behind this is simple. In the Wealth of Nations (1776), Adam Smith [31] already noticed the productivity advantage of cities. Since then, many studies have demonstrated that large cities are associated with greater productivity of firms and workers [32–34]. It is therefore natural to expect that greater urban concentration can be a consequence of industries achieving greater returns of productivity [35, 36]. Despite its theoretical appeal, and the cumulating evidence of increased labor productivity by agglomeration in Europe and China [22, 37, 38], few empirical analyses directly examine it, and thus this seemingly apparent association of scaling and returns to agglomeration actually remains unverified.

To be clear, the fact that concentration and agglomeration bring various productivity benefits for individual manufacturing sectors doesn’t help much to explain the inter-sector variation of their scaling behavior, because in theory all sectors could gain benefit from agglomeration. Thus, it is almost tautological to equate super-linear scaling with higher returns to agglomeration. Even if there is an association between scaling and returns, the direction could as well be the opposite. In fact, after we check this assumption in our result, we find that concentration promotes returns in almost all manufacturing sectors in the data, yet by various degrees. Those with greater agglomeration returns are not those with greater urban concentration.

Part of the difficulty to verify this relation lies in translating the conceptual idea of returns to agglomeration into an operational one in the urban context. To meet the challenge, we follow the wisdom in industrial economics to adapt a measure of scale elasticity into the urban context, and denote it as urban productivity. Scale elasticity is initially used in the analysis of firm-level production [39], but later on applied to the industry-level analysis of external economies of scale for manufacturing and trade at the industry and country level [40–42]. It deals with the relation between the intensity of labor/capital and the associated growth of output. Despite their conceptual difference, it has a very similar (Cobb-Douglas) form to the urban scaling equation and also displays a concise linear property, so is quite suitable for this analysis. Recently, scholars [43] use a similar approach to measure returns to agglomeration for U.S. trade industries and show that sector-wide returns to agglomerations are far from uniform.

On the other hand, recent advances in economic complexity and urban scaling offer us a different perspective on the urban growth of economic activities. The strand of literature in economic complexity holds that products are made not just by material but also with knowledge, and different products manifest different packages of knowledge [44]. The combination of existing components of knowledge is a key mechanism of knowledge creation [45]. Such combinatorial views of economic activities crystalize in the idea of knowledge complexity [46–49]—which distinguishes the level of specialization and diversification of knowledge embedded in economic activities and highlights the importance of complex interactions between various kinds of knowledge for continued growth. Meanwhile, recent literature on urban scaling examines the economic composition of cities, and proposes a strong positive relationship between city size and economic diversity [50]. Large cities have more diverse business establishments [51], the greater ability to make atypical combinations [52], and exhibit greater occupational and skill specialization that is less subjected to automation [53]. Since activities that are more complex, on average, are more specialized and hinge more on the combination of a diverse set of skills, they should be more favorable to urban concentration than activities that are less knowledge intense. In light of these, it is reasonable to assume that knowledge complexity can explain the scaling variation of manufacturing activities in China. Some related evidence of this relation was found for more general social-economic activities in the U.S. and in Brazil [49], which adds to the soundness of this assumption.

A remaining puzzle of complexity-based reasoning is the underlying relation between knowledge complexity and returns to agglomeration. It is unknown whether the complexity-scaling relation, given it exists, is mediated by returns to agglomeration or not. Indeed, literature on complexity tends to elude this issue and adopt a convenient strategy, assuming that complexity is associated with productivity, which in turn manifests its own importance. Yet as we have argued above, the inter-sector variation cannot be reasonably explained by intra-sector mechanisms. In the analysis, we focus on the inter-sector variation and endeavor to disentangle the relations between urban concentration, urban productivity and knowledge complexity. Our finding shows that, at the sector level, complexity actually is not positively associated with returns to agglomeration for manufacturing.

## Data and methods

China is known as the “world’s factory”, and manufacturing has long been a major source of the wealth of cities in China [28]. Existing studies on urban scaling in China have mostly examined scaling in a general manner [23, 25–27], without paying too much attention to the more fine-grained level of manufacturing. To address this lacuna, our analysis focused on manufacturing and obtained a complete set of 31 manufacturing sub-industries, from the traditional sectors such as clothing, and wood processing to modern sectors like electric equipment and computer manufacturing.

In particular, we analyze the urban scaling, returns to agglomeration, and complexity using sector-wide economic variables in the Bulletin of the Fourth National Economic Census (BFNEC). Because the study of scaling also requires population data, we collect prefecture-level city population from the Seventh National Population Census (SNPC), which is the most recent publicly available census of population in China.

The Bulletin of the Fourth National Economic Census is a comprehensive survey conducted nationwide from 2017 to 2019. The census was published separately on each prefectural city government’s website since 2020. We manually fetched all available files online and combined them into a single dataset. The resulting dataset contains economic indicators of registered “legal units”(mostly firms) in 31 sectors of manufacturing enterprises in 288 prefecture-level cities in China. We use the “annual revenue” at the prefecture-city level as the measure of socio-economic outputs for each sector. While the Gross Domestic Product (GDP) is more frequently used in previous studies, the annual revenue is highly correlated with GDP and is readily available in the Economic Census. Other key variables include the employment size of legal person units, the annual revenue of legal person units, R&D expenditure of legal person units, and the ratio of R&D expenditure to revenue.

We also obtained urban population data from the Seventh National Population Census, the results of which are published separately by each city government since 2020. A few cities didn’t publish their census online, so there are a few missing cases. Two estimates of the urban population are available in these official statistics—urban registered population under Hukou and urban permanent residents. Following Zünd and Bettencourt [54], we are aware of the huge migration issue in China (which makes the registered population highly biased) and thus consider the latter as a more accurate estimate of the urban population. After filtering out missing values, 205 cities and 30 manufacturing sectors (excluding “chemical raw materials and chemical products manufacturing”) were eventually included in the final analysis.

### Measure

First of all, we argue that the scaling of input (such as labor, resource) and output (number of products, revenue) should be distinguished. The larger scaling of the input doesn’t necessarily equal to greater scaling of the output, and vice versa. Existing studies often blur such boundaries. For example, in Bettencourt’s original paper “the origin of scaling in cities”, economic outputs (such as GDP) are simply grouped in the section of “socioeconomic rates” along with establishments and employment in their paper. Likewise, Balland and collaborators [49] treated the scaling of employment and the scaling of GDP and patent number similarly as metrics of urban concentration in their empirical analysis.

#### Urban concentration

We define the scaling of industrial employment (as urban population increases) as urban concentration. Following previous studies [23, 51, 55–58], we assume the following urban scaling model of employment size (*E*_*i*_) and revenue (*R*_*i*_) for industry *i*, where (*N*) is the urban population and *E*_0_ is the intercept.

$$\begin{array}{c} E_{i}=E_{0}N^{\beta_{concentration}} \end{array}$$ (1)

#### Urban productivity

For returns to agglomeration, we identify the scaling relation between industrial output and industrial labor employment as urban productivity. This relation is also measured in a similar Cobb-Douglas form as above and is very similar to scale elasticity in industrial economics [39–42] but adapted into the urban context. We demonstrate that scale elasticity is a suitable measure since it stays nearly constant at each employment scale. The relation between industrial revenue (*R*_*i*_) for industry *i* and employment *E*_*i*_ can be modeled as below, where *β*_*productivity*_ is the scaling component or scale elasticity, indicating levels of returns to agglomeration, and *R*_0_ is the intercept characterizing the baseline output level.

$$\begin{array}{c} R_{i}=R_{0}E_{i}^{\beta_{productivity}} \end{array}$$ (2)

Putting together, a combination of urban concentration and urban productivity would give us the urban scaling of the industrial output, as can be seen after substituting *E*_*i*_ in Eq 1 by Eq 2

$$\begin{array}{c} R_{i}=k_{0}N^{\beta_{concentration}\beta_{productivity}} \end{array}$$ (3)

where $k_{0}=R_{0}E_{0}^{\beta_{productivity}}$ is a constant.

#### Knowledge complexity

Complexity is measured here using the mean ratio of Research and Development (R&D) expenditure in each industry among cities. The reason is that knowledge complexity is associated with cumulative technological advances and the process of innovative creation. R&D expenditure is thus a good reflection of industrial-level knowledge creation through combination and is widely used in characterizing levels of technology in previous studies. It is thus reasonable to assume that complex industries need more R&D activities, which is consistent with previous studies [23, 59]. We also follow Balland [49, 60] to check the vintage of the patent categories of manufacturing industries as an alternative measure of complexity (see S3 Fig). Although the industry category for patents is not exactly the same as here, it is not difficult to eye bow that manufacturing sectors with higher R&D expenditure ratio correspond to those with more recent years of subclass introduction in patent systems.

These measures allow us to check the following assumptions in the analysis.

*H1: urban concentration is positively associated with urban productivity*

and

*H2: urban concentration is positively associated with knowledge complexity*

## Results

**Fig 1:**
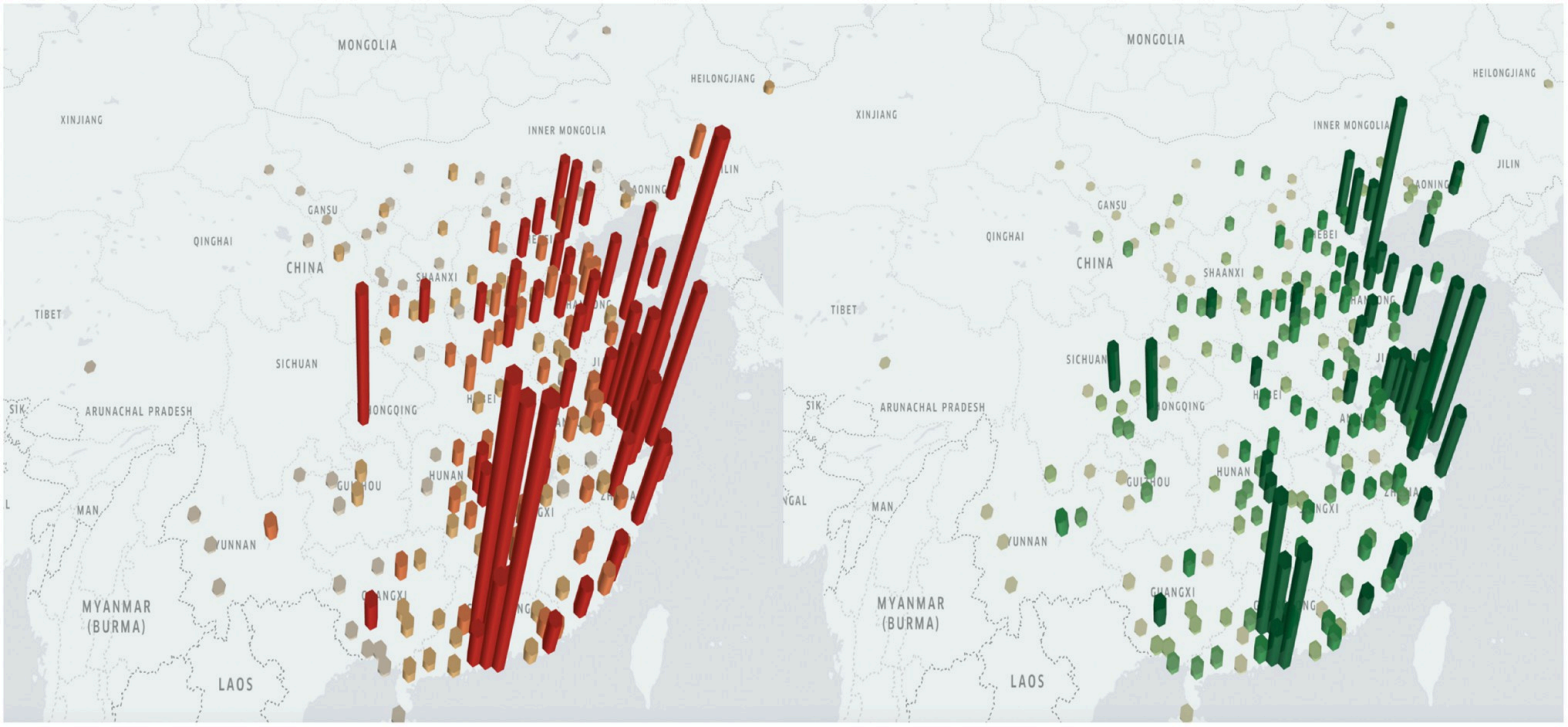
Maps of the spatial distribution. of (Left) industrial employment and (Right) industrial revenue in China’s prefecture-level cities.

The spatial distribution of manufacturing activities is highly unequal in China, as most inputs and outputs concentrate on the southeast coast of the country, particularly in the Yangtze River Delta, the Pearl River Delta, and the Beijing-Tianjin-Hebei region. The spatial bar plot in Fig 1 visualizes such inequality for total industrial employment and industrial revenue according to the statistics from BFNEC. Higher and darker-colored bars correspond to greater employment (Left) or output (Right). The employment distribution correlates with that of revenue but the two do not match exactly. The spatial inequality also seems more significant for employment than for revenue. Despite these natural-level industrial agglomerations [27] though, manufacturing activities are also widely scattered across the country.

**Fig 2:**
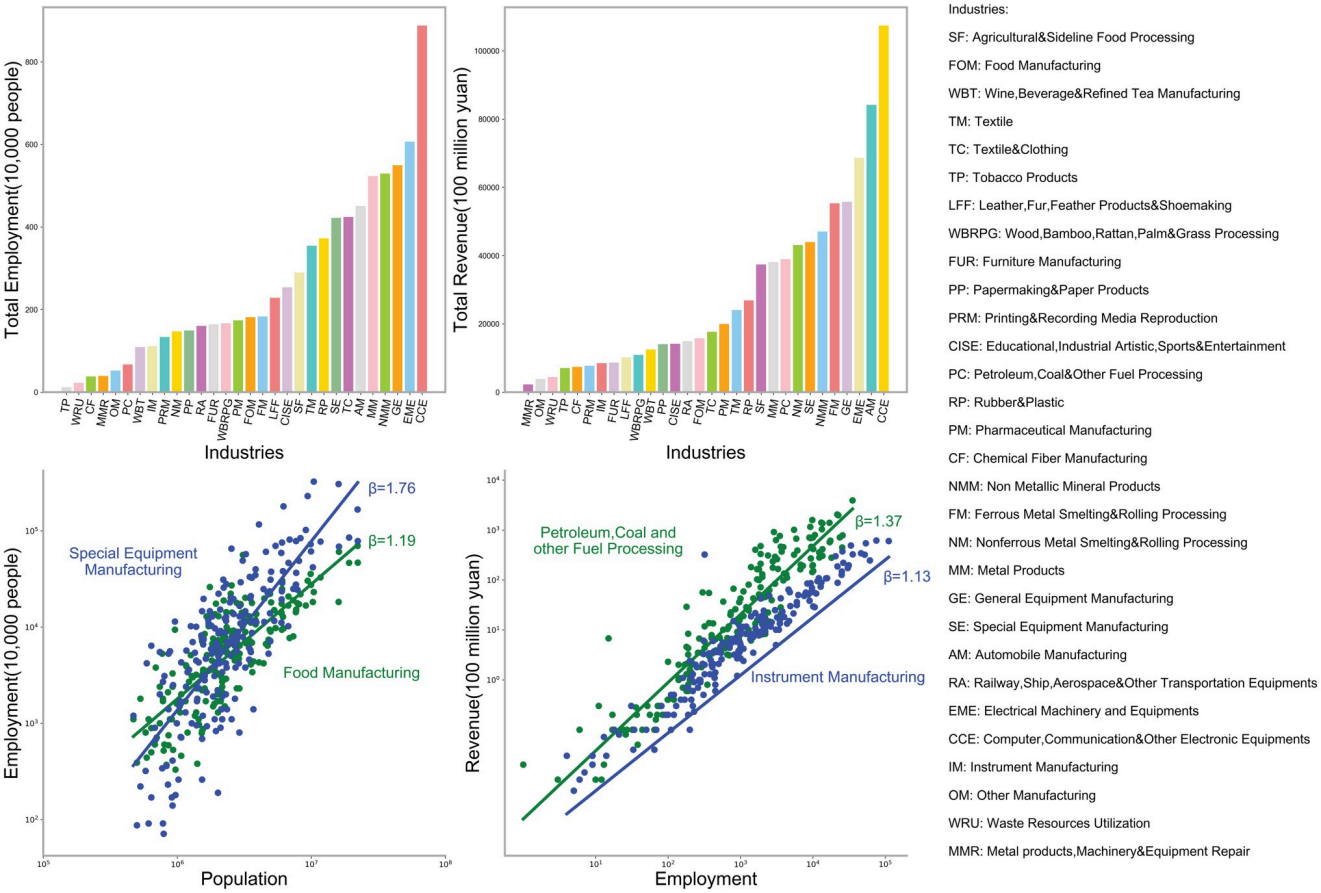
Urban scaling of manufacturing activities. (A) Total employment by industries and (B) Total Revenue by industries. Scaling relations between (C) urban population and industrial employment for “food manufacturing” and “special equipment manufacturing”. (D) industrial employment and annual revenue for “petroleum, coal, fuel processing” and “instrument manufacturing”.

Besides histograms of industrial employment (A) and revenue (B), Fig 2 provides examples of scaling laws for both “urban concentration” and “urban productivity”. As for urban concentration in (C), “special equipment” concentrates more in large cities (*β* = 1.76, *p* < = 0.001) than “food manufacturing” (*β* = 1.19, *p* < 0.001). Scaling results of urban productivity in (D) show that the revenue of “petroleum, coal and fuel processing” scale faster (*β* = 1.37, *p* < = 0.001) than that of “instrument manufacturing” (*β* = 1.13, *p* < = 0.001), indicating that the former has greater returns to agglomeration. (See more detailed results for all sectors in S1 Fig). Next, we associate urban concentration with returns to scale to see whether the former can be explained by the latter.

**Fig 3:**
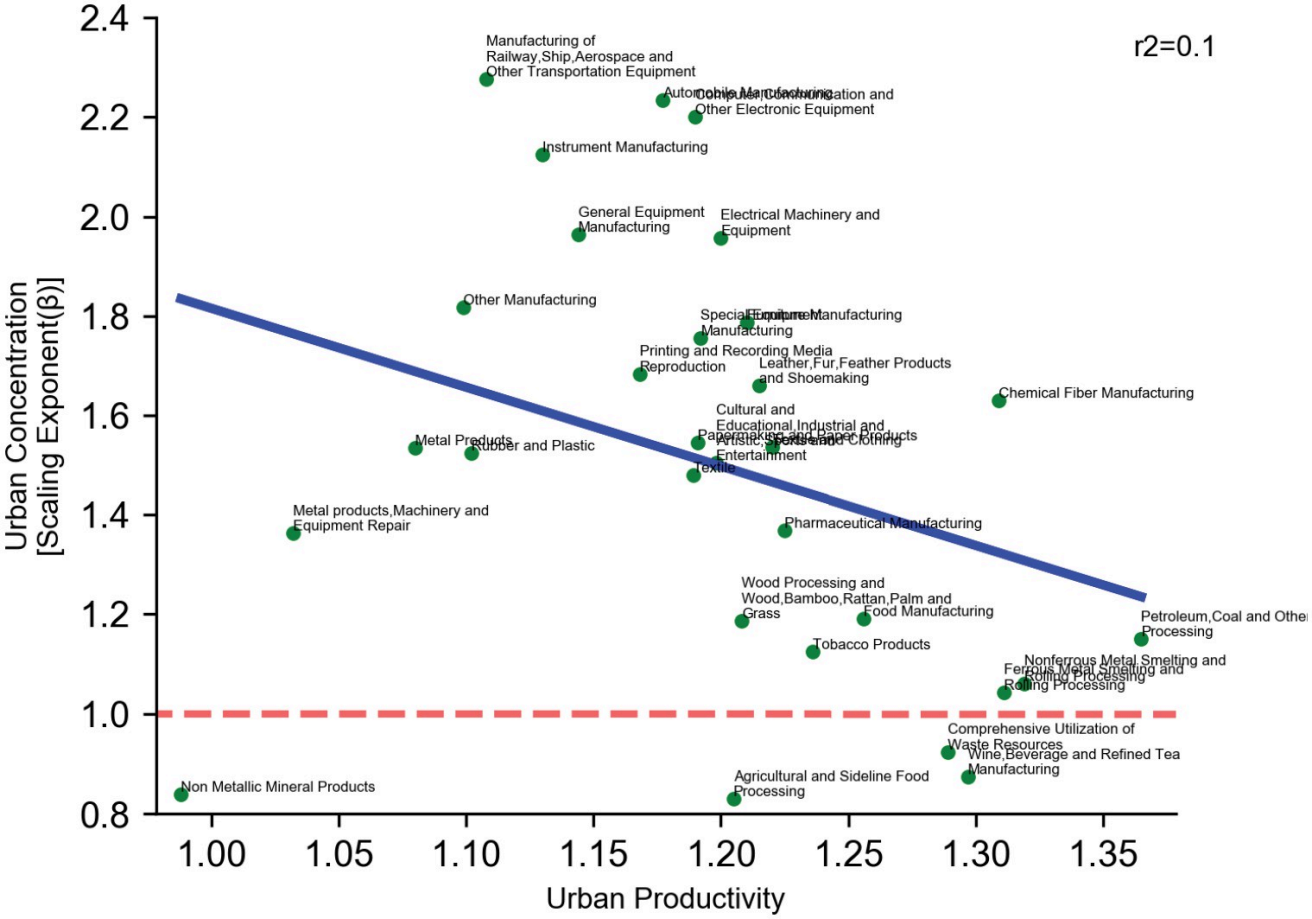
Urban concentration & urban productivity. The x-axis denotes urban productivity (measured by the scale elasticity). Urban concentration (measured by the scaling exponent of employment) slightly decreases with urban productivity. The blue solid line is a linear fit of the data. The red dashed line separates super-linear scaling from sub-linear scaling. Urban productivity measures returns to agglomeration by looking at the scale elasticity (scaling between employment and revenue).

How can the sector-level variation of urban concentration be explained? In Fig 3, we examine the relationship between urban productivity and urban concentration for each manufacturing sector. We find that our H1, which is based on conventional wisdom, is not supported. In particular, a linear fit clearly shows that the two are not positive but slightly negatively associated, although the relationship is not significant (*p* > 0.1, R-square around 0.1). Notably, industries like smelting, wine & beverage, and rolling have high scale elasticity but low urban concentration, while industries of instrument, equipment, ship & railway have low scale elasticity but high urban concentration. This result, however, should not be mistaken for the absolute value of scale elasticity. Note that the scale elasticity values on the urban productivity axis are above one in almost all industries (except for non-metallic mineral products, which display both low returns to agglomeration and low concentration), meaning that inside each sector, productivity still increases with scale. On the basis of this result, we conclude that urban concentration cannot be explained by the degree of returns to agglomeration.

**Fig 4:**
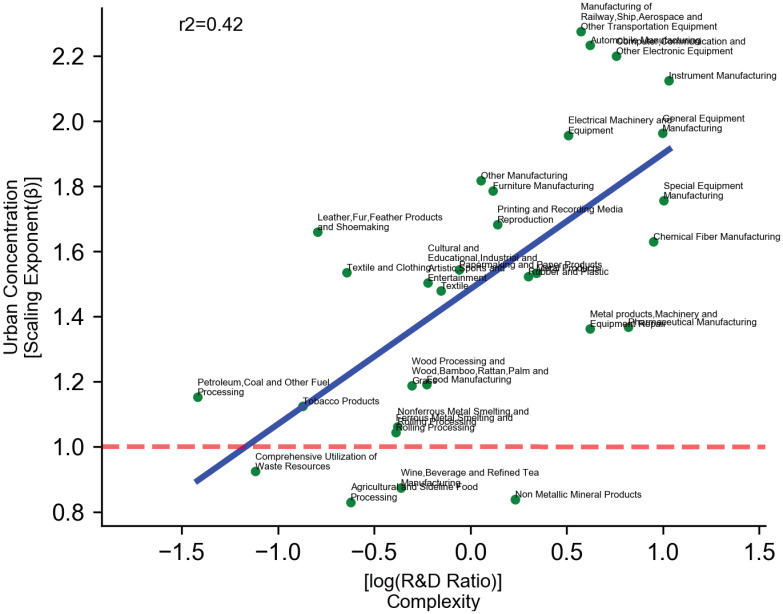
Urban concentration & complexity. The x-axis denotes the complexity of a sector. Complexity is measured here as the natural logarithm of the ratio between R&D expenditure and revenue. A higher value indicates greater knowledge complexity. The y-axis denotes urban concentration (measured by the scaling exponent of employment). Urban concentration increases for more complex manufacturing industries. The red dashed line separates super-linear scaling from sub-linear scaling.

If urban concentration cannot simply be explained returns to agglomeration, what could be the cause of its difference? We establish the relationship between knowledge complexity and urban concentration by looking at the scaling exponents for 30 manufacturing industries of different complexities. Fig 4 shows the results. Complex sectors like General Equipment Manufacturing have on average a high R&D expenditure ratio (2.71%) and its employment scales with 1.96. In the least complex sectors (such as Petrol & Fuel processing and Waste Resource Utilization) the ratios are merely 0.24% and 0.33%, and their employment scale with 1.15 and 0.92. In general, we find a significant pattern that complexity of industrial activities leads to greater concentration (Pearson’s r = 0.65, *β*=0.416, R-square = 0.42, *p* < 0.001). This result supports the knowledge complexity perspective, indicating that diversity matters more than efficiency for urban concentration. In addition, previous studies only distinguish between super-linearly scaling and sub-linearly scaling activities. Our result encompasses both and can further explain the variation inside the super-linear region. For instance, both Wood Processing (*β* = 1.19) and Paper Products (*β* = 1.55) scale super-linearly, yet the difference in their scaling exponents can be associated with different levels of complexity. The regression results of both *H1* and *H2* can be found in Table 1.

**Table 1:** Regression results: Effects of urban productivity and complexity on urban concentration.

	Model 1 Urban Productivity	Model 2 Knowledge Complexity
Coefficient	−1.586	0.416***
*R* ^2^	0.102	0.42
95% Interval	[−3.41, 0.24]	[0.23, 0.6]
Pearson r	−0.32	0.65
N	30	30

Notes.

**:*p* < 0.01,

***:*p* < 0.001 (two-tailed test).

**Fig 5:**
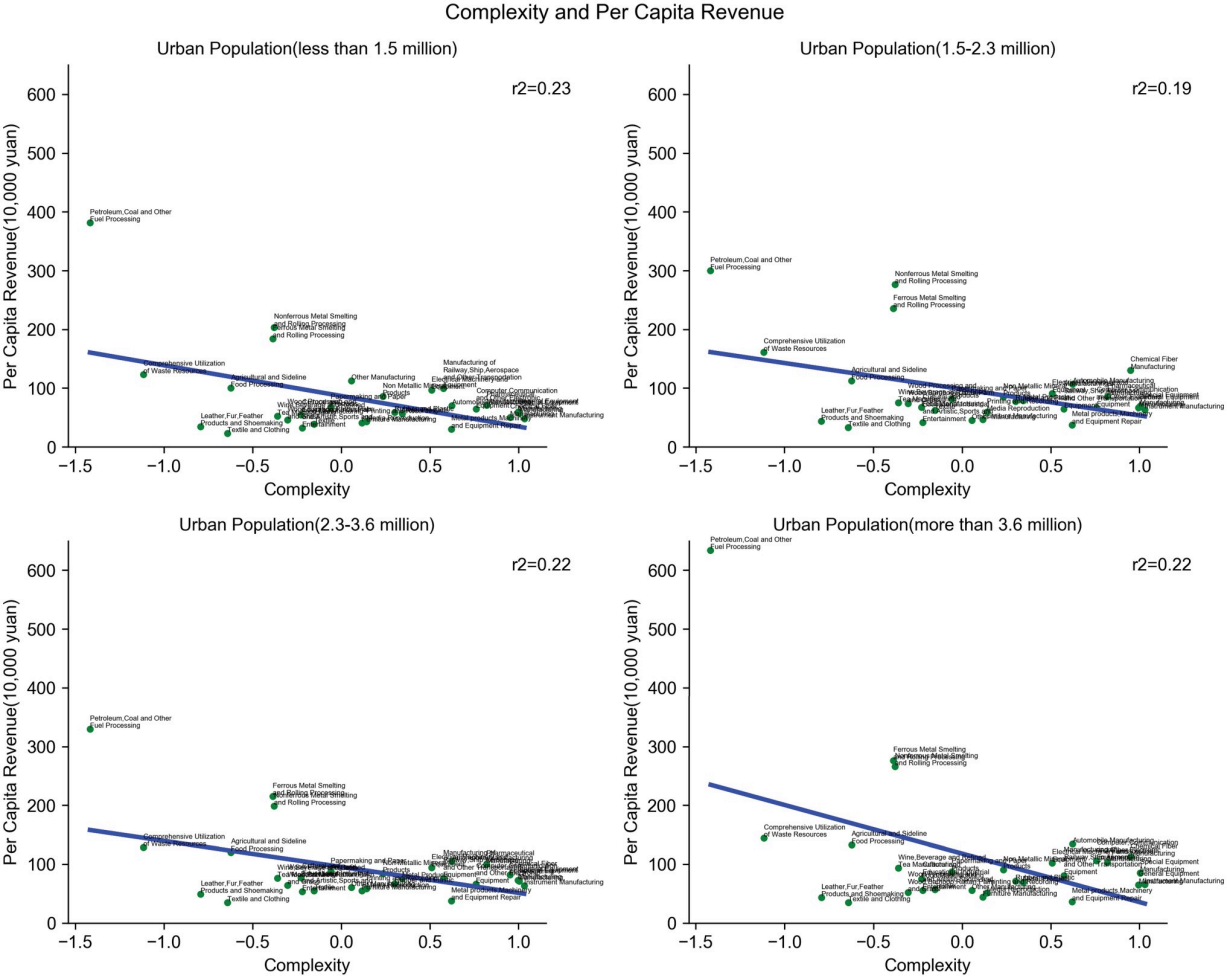
Complexity and average per capita revenue of manufacturing industries by city group. Complexity in the x-axis is measured in the same way as in Fig 4 using the natural logarithm of the ratio of R&D expenditure. Cities are divided into four cities groups. For each industry, we average the Per Capita Revenues for cities within the same group. In the four city groups (urban population “below 1.5 million”,“1.5–2.3”, “2.7–5.4” million, and “5.4 million and above”), the Per Capita Revenue tends to decrease slightly as complexity increases.

**Fig 6:**
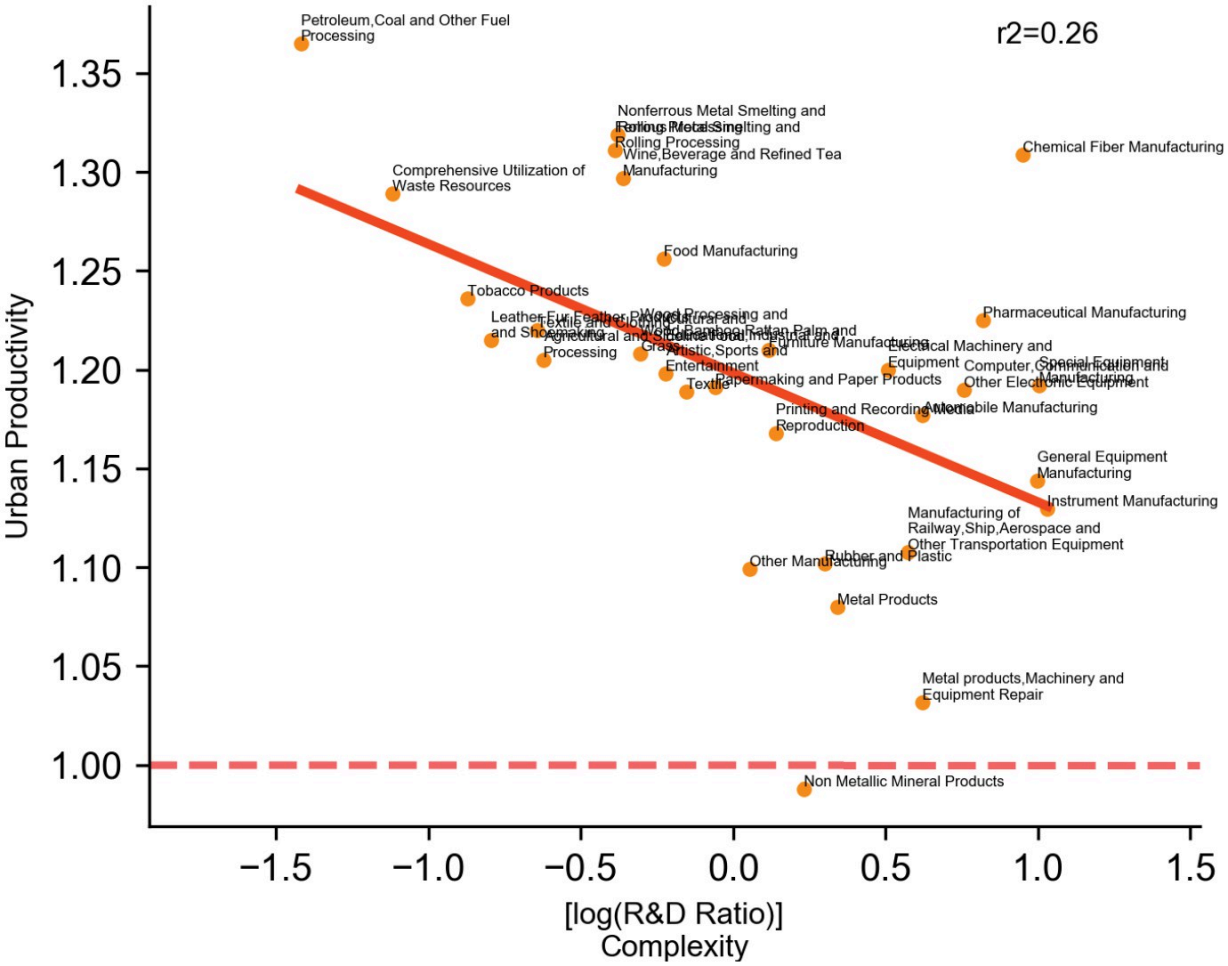
Complexity & urban productivity. Complexity is measured in the same way as in Fig 4. Urban Productivity is measured in the same way as in Fig 3.

Besides urban scaling, we further look closely at the average per capita revenue for industries of varying complexities. We first assign cities into four groups by their population size. In each group, we check the relation between per capita revenue and complexity. The result in Fig 5 shows that, in all four city groups, per labor revenue tends to slightly decrease with complexity. In particular, the intuitively least knowledge intense industries (such as petro&gas processing, utilization of waste resources, and metal smelting) are found to have the highest per labor revenues. Among the three city groups, the slopes also become steeper as population scales, which indicates that the gap of per labor revenue might become enlarged in larger cities. In light of this, we investigate whether complexity is negatively associated with returns to agglomeration, and the result is significant (*p* < 0.01, R-square = 0.26) as shown in Fig 6.

## Conclusion

Cities are known to be centers of creativity [52, 61]. In “Growth, innovation, scaling, and the pace of life in cities”, Bettencourt [23] demonstrates the dramatic difference “between growth fueled by innovation versus that driven by economies of scale”. Here in this article, we further this inquiry by looking beyond the divide between super-linear and sub-linear scaling activities and seek to explain the variation of urban scaling for fine-grained production activities.

In particular, this article provides an approach to demonstrating the relations between urban scaling, returns to agglomeration, and knowledge complexity. Our findings are twofold. First, we prove that, rather than being a positive predictor, returns to agglomeration are slightly negatively associated with urban scaling rates. Second, we measure the complexity for each sector and demonstrate that the variation of urban concentration can be largely explained by their complexity, consistent with the knowledge complexity perspective. Specifically, complex manufacturing sectors are found to concentrate more in large cities than less complex sectors in China. This result provides support for the view that the growth of complex activities hinges more on diversity than on efficiency. It also complicates existing interpretation of the “complexity—concentration” relation which emphasizes greater performance, increasing returns, or competitive advantage. Indeed, it may also remind readers of the traditional wisdom in organizational studies [62, 63], which abandons perfect functionality and emphasizes entrepreneurial opportunities, and in cultural anthropology [64–66], which treats labor densification as a more general cultural form of “involution” that may result in a stasis of productivity. Greater concentration doesn’t necessarily rely on the performance or productivity [67, 68]. Concentration tends to happen as long as the perceived entrepreneurial opportunities keep attracting more and more new entrepreneurs to initiate their business in the condensed area, which may even make the failure rates higher in the region than elsewhere in the short run. Bridging these ideas with recent wisdom in knowledge complexity, we suggest that instead of performance, social-political factors [69] highlighting entrepreneurial opportunities for combinations of diverse knowledge components play more important roles in shaping the geographic features of the product economy.

Finally, scaling properties in different systems might be different [70] and subjected to path-dependency [71]. Our findings are also helpful for tracking and understanding differences in urban complex systems, given the fact that the grand pattern of urban concentration may usually seem isomorphic. The findings that returns to agglomeration is not the driving force of urban concentration of manufacturing activities in China, for example, contrast with what would be found in pure knowledge systems such as patents in the U.S. (See S4 Fig). The case here can be juxtaposed in future studies with other cases for better comparison.

## Supporting information

S1 Fig Urban productivity. The scaling relationship between employment and revenue for manufacturing sectors indicates urban productivity. In each subgraph, the x-axis is the total size of employment in the manufacturing sector (in 10,000), and the y-axis is the total annual operating income (in 100 million yuan). The black dots are cities, the blue line is the fitted line between the number of employees and the total annual revenue. There is a significant linear relationship (Most R-square is greater than 0.8).
(TIF)

S2 Fig Urban concentration. The scaling relationship between the urban population in each city and the number of persons employed in manufacturing units. In each subgraph, the x-axis is the urban population (10,000 people), the Y-axis is the manufacturing employment (10,000 people). Black dots refer to cities, the blue line is the fitted linear curve between urban population and manufacturing employment. The results show that there is a significant linear relationship between the number of employment in each type of manufacturing and the urban population. With the growth of the urban population, employment growth in most manufacturing sectors has been superlinear.
(TIF)

S3 Fig Measures of complexity. Comparison of the R&D measure of complexity with Alternatives. (A). Knowledge complexity and scaling of patent numbers in China, which is similar to previous studies. (B). Knowledge complexity and scaling of revenue for manufacturing sectors. The terms show that sectors that have a higher R&D expenditure ratio correspond to those with higher complexity (greater average years of subclass introduction) in patent systems.
(TIF)

S4 Fig Comparison with U.S. patent systems. Relations of Urban concentration and Urban productivity for the U.S. patent systems. For comparison, this graph plots the urban concentration and urban productivity relation for the U.S. patent system, in which greater concentration is positively associated with greater productivity (returns to agglomeration).
(TIF)
