# Supplementary figures and images for: Understanding urban concentration of complex manufacturing activities in China
Source: PLoS One. 2023 Mar 16;18(3):e0278469. doi: 10.1371/journal.pone.0278469 (PMC10019714; doi:10.1371/journal.pone.0278469)

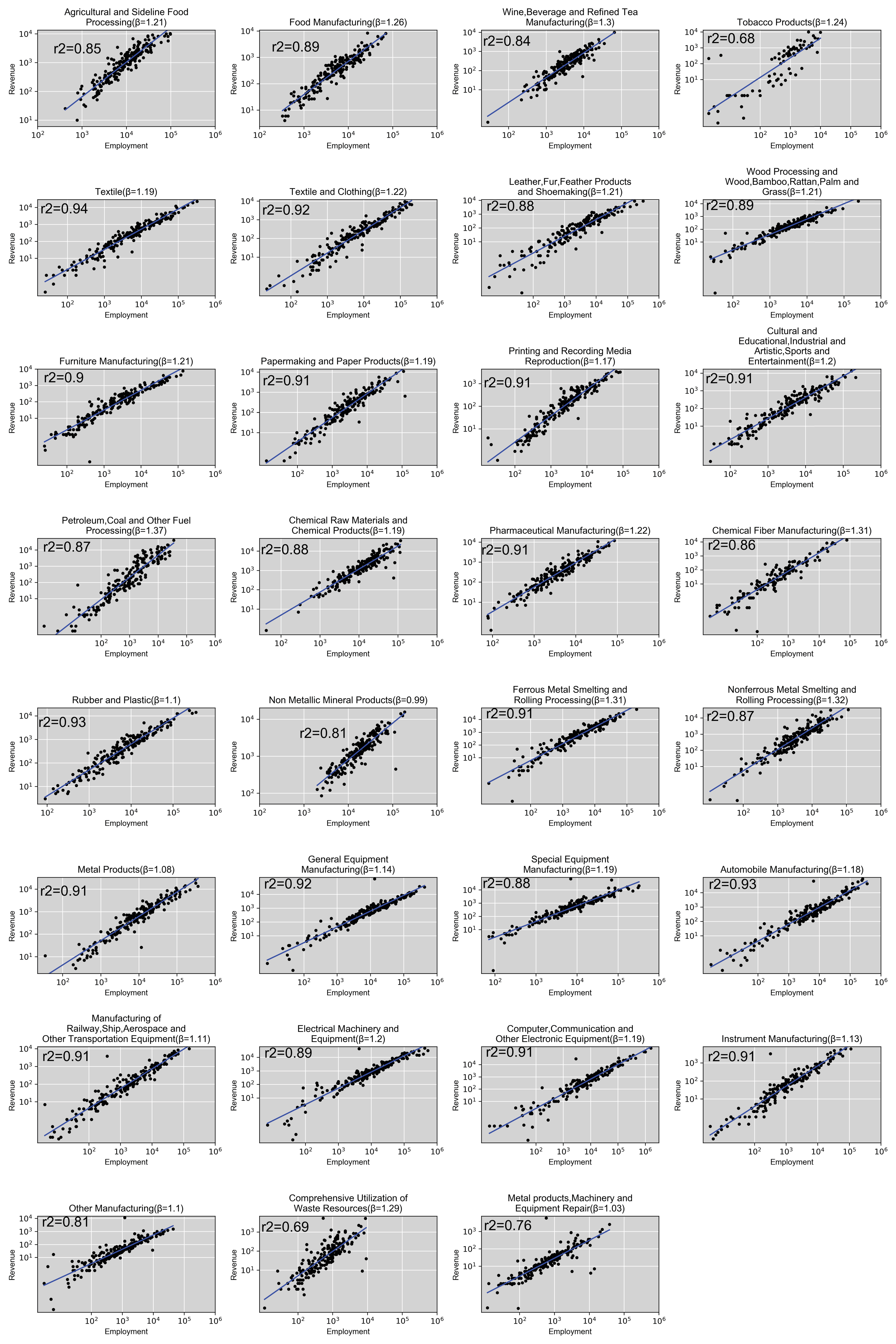

Supplement: S1 Fig — The scaling relationship between employment and revenue for manufacturing sectors indicates urban productivity. In each subgraph, the x-axis is the total size of employment in the manufacturing sector (in 10,000), and the y-axis is the total annual operating income (in 100 million yuan). The black dots are cities, the blue line is the fitted line between the number of employees and the total annual revenue. There is a significant linear relationship (Most R-square is greater than 0.8). (TIF) [file pone.0278469.s001.tif]

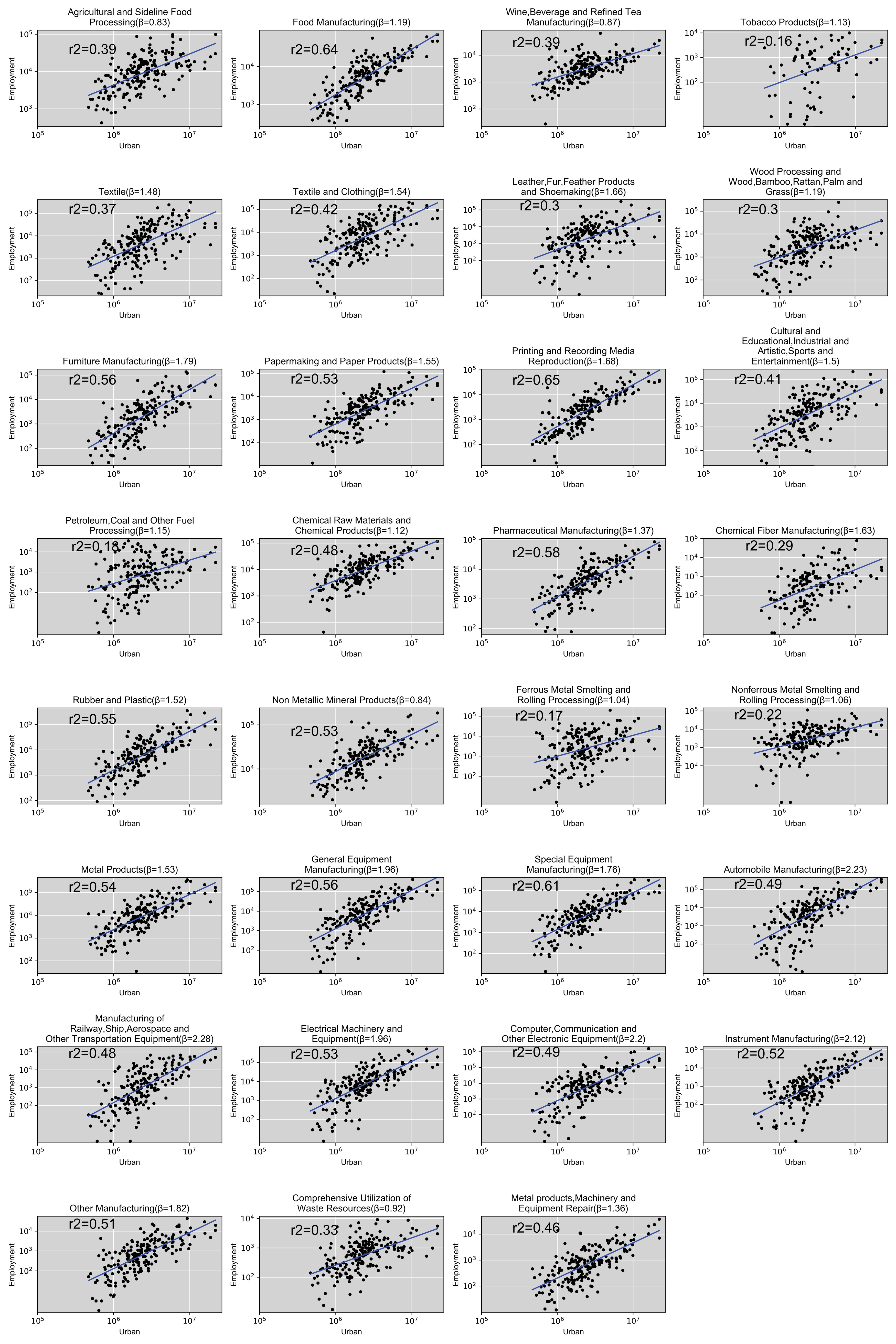

Supplement: S2 Fig — The scaling relationship between the urban population in each city and the number of persons employed in manufacturing units. In each subgraph, the x-axis is the urban population (10,000 people), the Y-axis is the manufacturing employment (10,000 people). Black dots refer to cities, the blue line is the fitted linear curve between urban population and manufacturing employment. The results show that there is a significant linear relationship between the number of employment in each type of manufacturing and the urban population. With the growth of the urban population, employment growth in most manufacturing sectors has been superlinear. (TIF) [file pone.0278469.s002.tif]

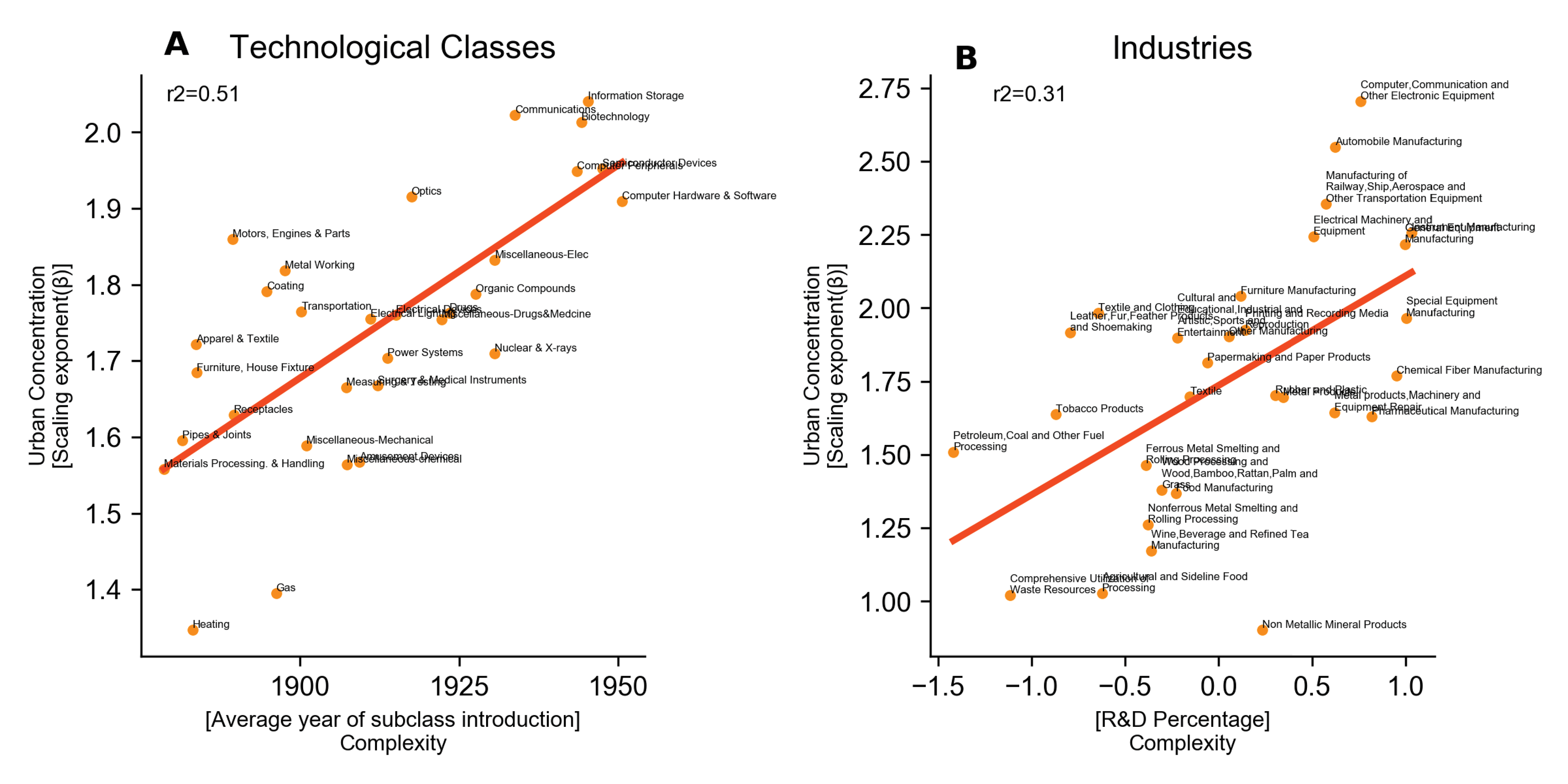

Supplement: S3 Fig — Comparison of the R&D measure of complexity with Alternatives. (A). Knowledge complexity and scaling of patent numbers in China, which is similar to previous studies. (B). Knowledge complexity and scaling of revenue for manufacturing sectors. The terms show that sectors that have a higher R&D expenditure ratio correspond to those with higher complexity (greater average years of subclass introduction) in patent systems. (TIF) [file pone.0278469.s003.tif]

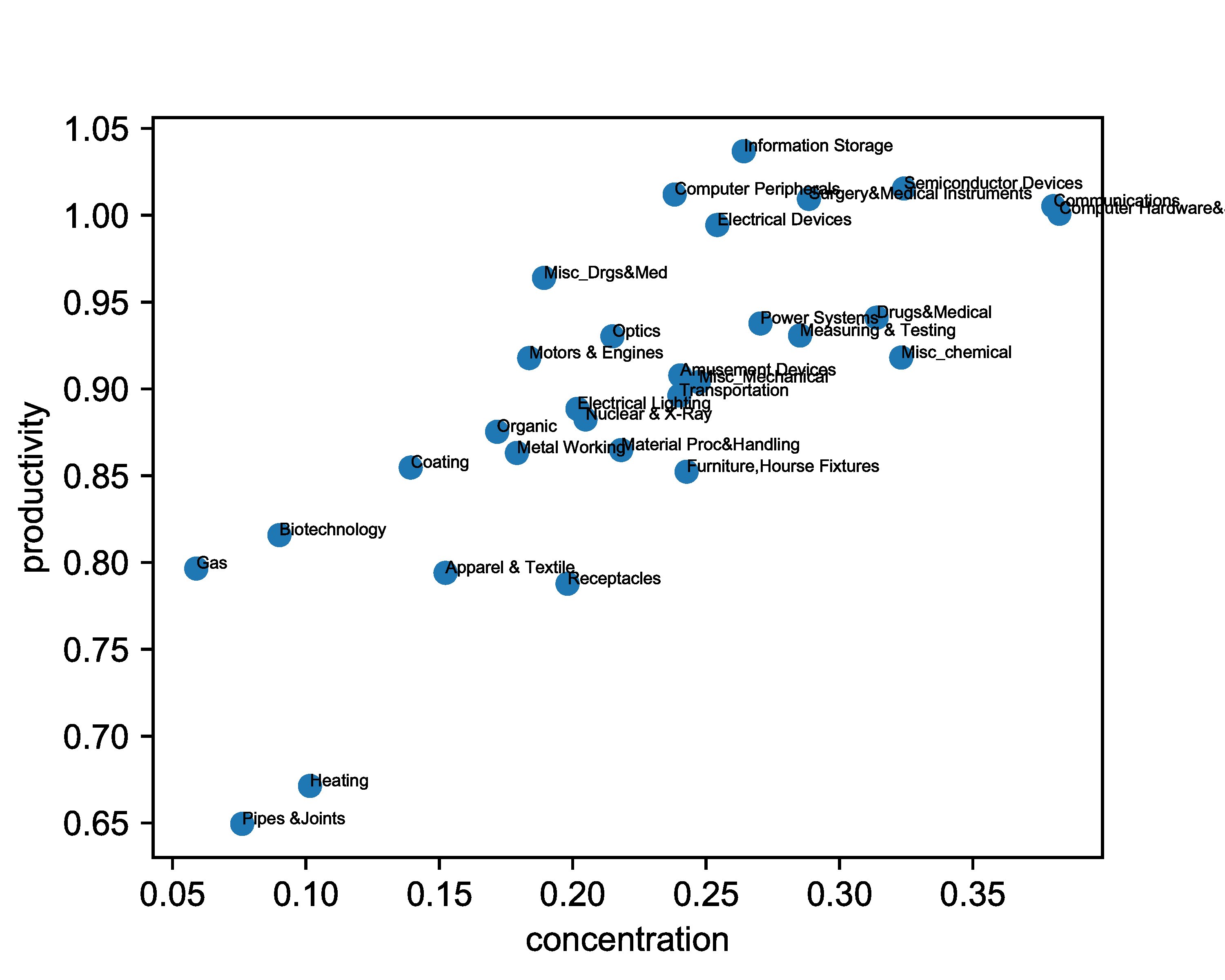

Supplement: S4 Fig — Relations of Urban concentration and Urban productivity for the U.S. patent systems. For comparison, this graph plots the urban concentration and urban productivity relation for the U.S. patent system, in which greater concentration is positively associated with greater productivity (returns to agglomeration). (TIF) [file pone.0278469.s004.tif]
